# Topical Collection on Sensors and Biosensors for Environmental and Food Applications

**DOI:** 10.3390/s26144625

**Published:** 2026-07-21

**Authors:** Álvaro Torrinha, Simone Morais

**Affiliations:** REQUIMTE/LAQV, ISEP, Polytechnic of Porto, Rua Dr. António Bernardino de Almeida, 431, 4249-015 Porto, Portugal

Sensor technology is critical to the perception of chemical data across diverse systems of interest. The environment and food are complex systems, and analyzing them is a very challenging task, though it is essential to safeguard ecosystems’ integrity, food quality, and ultimately “One Health”. Analytical techniques based on chromatography and mass spectrometry have been primarily applied for pollutants, contaminants, and nutrients analysis, though with inherent operability drawbacks. These can be potentially overcome through the involvement of interdisciplinary contributions spanning nanotechnology, biotechnology, and environmental sciences in the development of innovative sensing platforms featuring unique characteristics such as miniaturization, portability, facile and rapid operation, and sustainability. Nevertheless, several challenges continue to limit the widespread implementation of sensors. There is especially a need for improved robustness in complex matrices, standardized validation procedures, long-term stability, cost-effective manufacturing, integrative design, and field-deployable and continuous monitoring systems. Despite the excellent analytical performance under controlled laboratorial conditions, the transfer of many sensing platforms into routine environmental and food monitoring settings is rather difficult and remains limited.

Thus, the Topical Collection “Sensors and Biosensors for Environmental and Food Applications” gathers contributions that reflect current efforts to address some of these challenges and future perspectives of sensors and biosensors for environmental and food applications. The studies comprise original research (Contributions 1–13) and reviews/perspectives (Contributions 14 and 15). A large number of these studies focus on the analysis of contaminants and nutritional parameters in food (Contributions 1, 3, 5–7, 9 and 11) as these are closely linked to human health through their consumption, whereas environmental studies (Contributions 2, 8, 10 and 13) pursue a complementary and reciprocal objective—to inform on hazardous substances. Instead of focusing on a single sensing technology, the Collection explores a diversity of technological principles of analyses that now characterize the field of sensors. Electrochemical transduction is significantly explored given the simplicity, low cost, and miniaturization capacity (Contributions 6, 8–10, 12 and 13). Likewise, optical approaches including fluorescent biosensors (Contributions 3 and 5), spectroscopic techniques (Contributions 2 and 11), and colorimetric (Contribution 1) also demonstrate optimal sensing capabilities. However, future sensing systems may consist of the combination of multiple transduction mechanisms (Contributions 6, 10 and 15) to enhance analytical performance in different practical scenarios.

Additionally, this Collection also emphasizes the importance of the implementation of nanotechnological and biotechnological materials/entities on sensors as well as their combination for the analytical outcome. Carbon nanomaterials (Contribution 13), graphene derivatives (Contributions 5, 6 and 12), carbon fibers (Contribution 8), metallic nanoparticles (Contributions 3, 6, 9, 10 and 12), semiconductor composites, and metal–organic frameworks (Contribution 9) are demonstrated to be fine examples of how rational material architectures can positively impact the analytical signal. On the other hand, biosensing strategies, either by using aptamers (Contributions 5, 6, 9 and 13) or antibodies (Contribution 3) for the development of aptasensors and immunosensors, can effectively resolve selectivity issues in demanding matrices, with certain polymers having similar capabilities, as seen in molecularly imprinted polymer-based sensors (Contribution 12). These advances enable the effective detection of contaminants in environmental and food matrices including heavy metals (Contributions 2 and 10), pharmaceuticals (Contributions 8 and 10), aflatoxins (Contribution 14), fumonisins (Contribution 5), cyanotoxins (Contribution 13), bacterial pathogens (Contribution 9), and marine toxins (Contribution 12). Beyond these analytes, a broader scope of action has been investigated, namely, food quality (Contribution 7), nutritional evaluation (Contributions 1 and 3), and even consumer perception (Contribution 4).

Notably, the Collection also highlights contributions that promote a transition from laboratory instrumentation to portable and field-deployable sensing devices. The development of smartphone-assisted sensors (Contribution 1), portable Raman spectroscopy (Contribution 2), disposable electrochemical platforms (Contributions 8 and 10), extended-gate field-effect transistors (Contribution 13), and compact optical systems (Contribution 5) demonstrate ongoing efforts to move the analytical systems to point-of-need where rapid decision-making may be critical.

The contributions of this Topical Collection show that future progress will depend not solely on achieving lower detection limits but on developing integrated sensing ecosystems that combine advanced materials, intelligent data analytics, portable instrumentation, sustainability, and practical applicability. Ultimately, they will contribute to the advancement of analytical science, aligned with the One Health framework, recognizing that the quality and safety of environment and food systems are critical to ecosystems and human well-being.

We sincerely acknowledge all authors, reviewers, and academic editors who have ensured the rigor, quality, and timeliness of the contributions gathered in this topical collection. We hope that the outcomes of this Topical Collection will be of interest to the *Sensors* readership and to the broader community working at the interface of analytical chemistry, environmental monitoring, and food safety.

## Data Availability

No new data were created or analyzed in this study. Data sharing is not applicable to this article.

